# Transforming growth factor-beta1 suppresses hepatocellular carcinoma proliferation via activation of Hippo signaling

**DOI:** 10.18632/oncotarget.14523

**Published:** 2017-01-05

**Authors:** Xiaodong Zhang, Qing Fan, Yan Li, Zhaoguo Yang, Liang Yang, Zhihong Zong, Biao Wang, Xin Meng, Qin Li, Jingang Liu, Hangyu Li

**Affiliations:** ^1^ Department of General Surgery, The Fourth Affiliated Hospital of China Medical University, Shenyang, P. R. China; ^2^ Department of Oncology, Tumour Angiogenesis and Microenvironment Laboratory (TAML), The First Affiliated Hospital of Jinzhou Medical University, Jinzhou, P. R. China; ^3^ Department of Biochemistry and Molecular Biology, College of Basic Medical Sciences of China Medical University, Shenyang, P. R. China

**Keywords:** transforming growth factor-beta, hepatocellular carcinoma, Hippo signaling, proliferation

## Abstract

In this study, we examined the expression of core proteins of the Hippo signaling pathway in hepatocellular carcinoma (HCC) cells treated with transforming growth factor-β 1(TGF-β1) and investigated the relationship between TGF-β1 and the Hippo signaling pathway, in order to better understand their roles in HCC and their potential implications for cancer therapy. We prove that the Hippo signaling pathway is involved in the TGF-β1-induced inhibition of the growth of HCC cells. Large tumor suppressor expression (LATS1) was overexpression and yes association protein 1(YAP1) translocated from the nucleus to the cytoplasm in HCC cells treated with TGF-β1. Overexpression of LATS1 and the nucleocytoplasmic translocation of YAP1 play an anti-oncogenetic role in the occurrence and development of liver cancer. Our findings provide new insight into strategies for liver cancer therapy.

## INTRODUCTION

Hepatocellular carcinoma (HCC) is one of the most common malignancies and the second leading cause of cancer-related death in China [1]. Despite the new advances in cancer diagnosis and treatment, surgical reaction and liver transplantation are still the best options for treating HCC [2]. However, HCC is associated with poor prognosis and a high recurrence rate, and the underlying molecular mechanisms are still unknown. Therefore, improving our understanding of the underlying molecular mechanisms is critical for the establishment of therapeutic strategies and effective metastasis-targeted therapy.

Transforming growth factor-β1 (TGF-β1) is a multifunctional polypeptide cytokine that plays a role in inflammation, it is produced by macrophages, stromal cells and tumor cells [3–5]. Under hypoxic and inflammatory conditions, TGF-β1 plays a role in tumor progression by regulating a wide variety of cellular processes, such as cell proliferation, differentiation, migration, and apoptosis [6]. Recent evidence has demonstrated that TGF-β1 is a major tumor suppressor in premalignant cells, while in the late stages of cancer progression, TGF-β1 induces epithelial-mesenchymal transition (EMT) and leads to increased metastasis of cancer cells [7–9]. Because of the critical role of TGF-β1 in the process of metastasis, antagonists that target TGF-β1 signaling have been developed in cancer therapeutics [10]. However, clinical efficacy of TGF-β1-targeting drugs in cancer therapy has been limited. Considering the conflicting functions of TGF-β1 in cancer development, it is not surprising that general inhibition of TGF-β1 expression may accelerate the progression of preneoplastic lesions, in which TGF-β1 still acts as a tumor suppressor. Thus, it is essential to elucidate the changes of TGF-β1 during the process of cancer development and metastasis.

The Hippo signaling is a highly conserved tumor suppressor pathway that plays a key role in the regulation of organ size and cell proliferation and apoptosis [11, 12]. The core components of this pathway are MST1/2 (mammalian sterile-20 like 1 and 2), LATS1/2 (large tumor suppressor 1 and 2) and YAP (yes association protein) [13]. YAP is a candidate oncogene in many human cancers, when the Hippo signaling pathway is activated, YAP is phosphorylated by LATS1/2, and then restricted to the cytoplasm or degraded [14]. Recent studies have shown that YAP1 interacts with Smad to regulate cellular responses induced by TGF-β1 in stem cells [15]; however, Nallet-Staub et al. insist that cell–cell contact-driven activation of the Hippo pathway is ubiquitous and does not interfere with TGF-β1 signaling [16]. Therefore, it is not clear what roles of the Hippo signaling pathway and the TGF-β1 they play in the development and progression of HCC, as the findings reported so far are contradictory.

In this study, we examined the expressions of LATS1 and YAP1 in HCC cells treated with TGF-β1, and found that TGF-β1 inhibited the growth of HCC cells by activating the Hippo signaling pathway.

## RESULTS

### TGF-β1 inhibits *in vitro* growth of HCC cells

**Figure 1:**
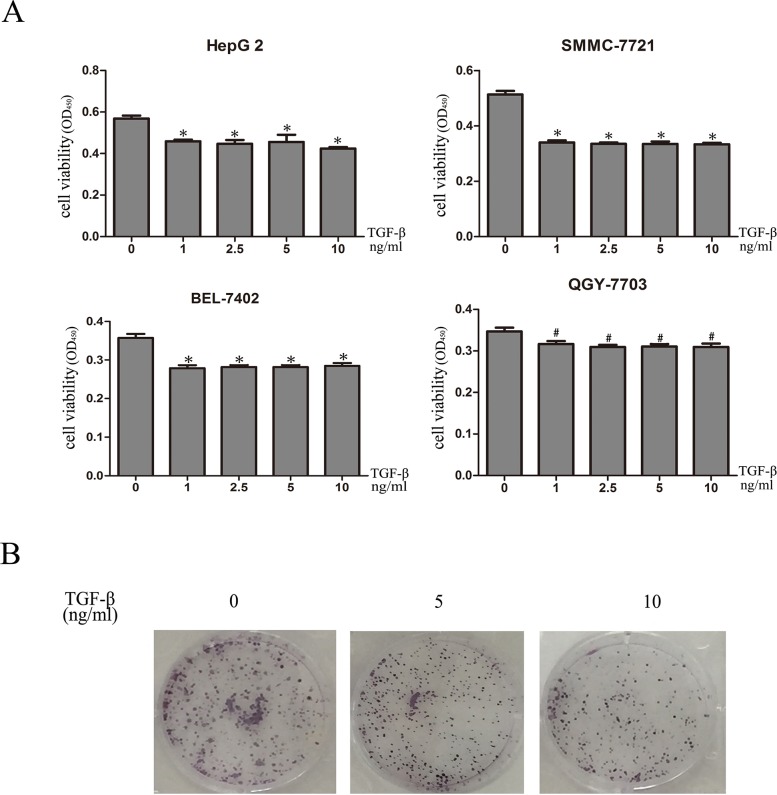
TGF-β1 inhibits *in vitro* growth of HCC cells **A**. SMMC-7721, Bel-7402, HepG2 and QGY-7703 cells were treated with 0, 1ng/mL, 2.5ng/mL, 5ng/mL and 10ng/mL TGF-β1 for 48 hours. Cell viability was determined by CCK-8 assay. **B**. 0, 5ng/mL and 10ng/m TGF-β1 treated cells formed fewer colonies compared with those cells without TGF-β1. Data are expressed as the mean ± SEM of three independent experiments, *P<0.05, #P>0.05.

In order to investigate the biological role of TGF-β1 in HCC, we assessed the effects of TGF-β1 on the viability of SMMC-7721, Bel-7402, HepG2 and QGY-7703 cells. The results obtained from the CCK-8 assay confirmed that the growth of SMMC-7721, Bel-7402, HepG2 and QGY-7703 cells was clearly inhibited by TGF-β1 (Figure 1A). And colony formation showed that TGF-β1 treated cells formed fewer colonies compared with those cells without TGF-β1 (Figure 1B).

### TGF-β1 suppresses the HCC cells proliferative capacity but does not promote apoptosis

**Figure 2:**
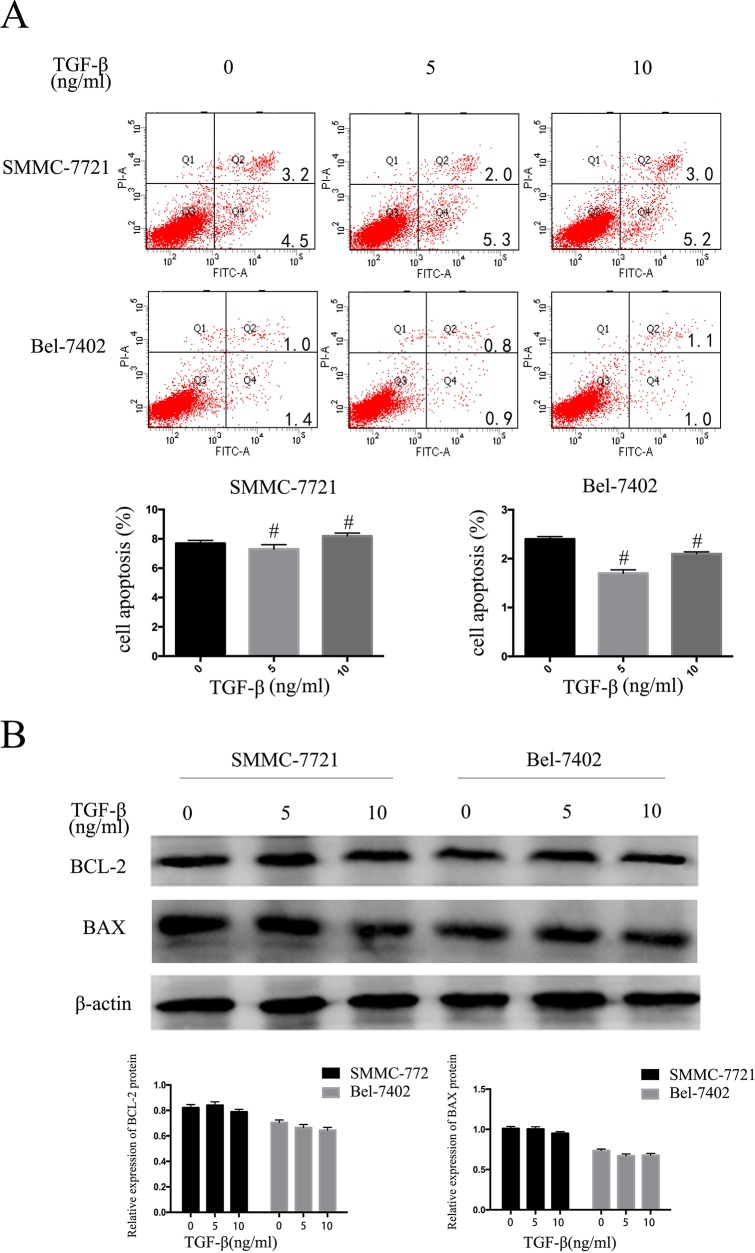
TGF-β1 suppresses the HCC cells proliferative capacity but does not promote apoptosis SMMC-7721 and BEL-7402 cells were treated with 0, 5ng/mL and 10ng/mL TGF-β1 for 48 hours. **A**. apoptosis **B**. western blotting examined the expression levels of Bcl-2 and Bax. Data are expressed as the mean ± SEM of three independent experiments, #P>0.05.

We then performed apoptosis assays in HCC cells after 48 hours treated with 5ng/mL or 10ng/mL TGF-β1. Apoptosis assays showed that cell apoptosis rates had no clearly change compared with control cells (P > 0.05; Figure 2A). We then performed Western blotting to measure Bcl-2 and Bax protein expression levels, while Bcl-2 and Bax demonstrated no significant differences in expression (Figure 2B). These results indicate that TGF-β1 suppresses the HCC cells proliferative capacity but does not promote apoptosis.

### TGF-β1 activates the Hippo signaling pathway in HCC cells

**Figure 3:**
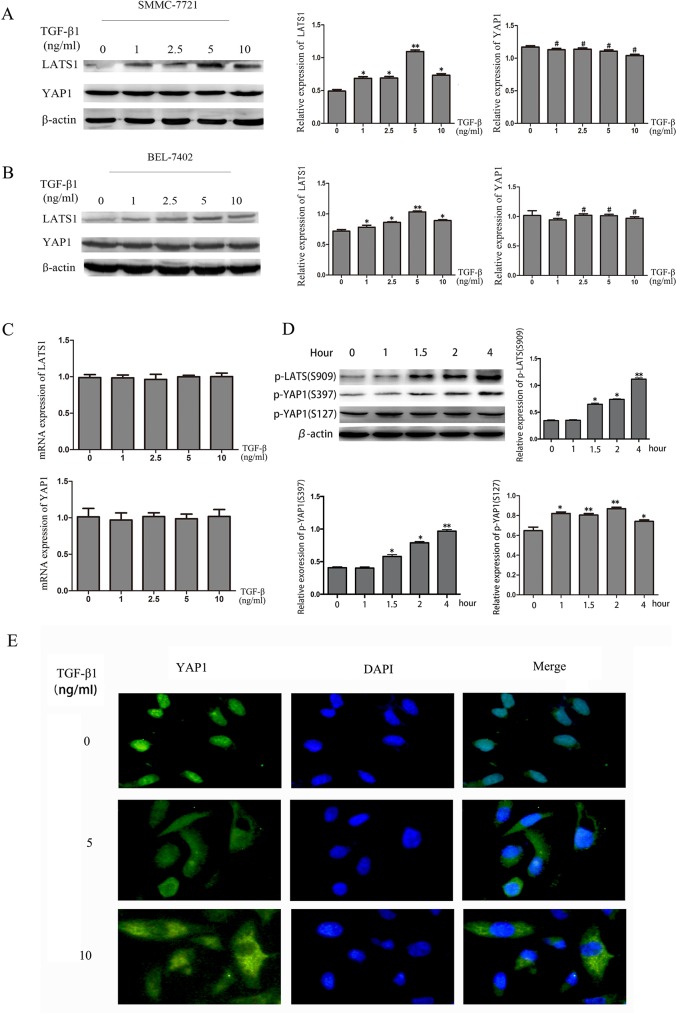
TGF-β1 activates the Hippo signaling pathway in HCC cells **A, B**. The protein levels of LATS1 and YAP1 were measured by western blotting in HCC cells treated with 0, 1ng/mL, 2.5ng/mL, 5ng/mL and 10ng/mL TGF-β1 for 48 hours. **C**. YAP1 mRNA levels were quantified using real-time PCR. **D**. The protein levels of p-LATS1(S909), p-YAP1(S397) and p-YAP1(S127) were measured by western blotting in HCC cells treated with 5ng/mL TGF-β1 for 1h, 1.5h, 2h and 4h. **E**. the immunofluorescent staining of YAP1 in SMMC-7721 cells treated with 0, 5ng/mL and 10ng/mL TGF-β1 for 48 hours. *P<0.05, **P<0.01, #P>0.05.

Activation of the Hippo signaling pathway leads to high expression of LATS1 and YAP1 phosphorylation on specific serine residues [17]. Thus, we investigated the expression level of LATS1 and YAP1 in SMMC-7721 and Bel-7402 cells, which were treated with TGF-β1. The western blotting showed that LATS1 expression was increased in SMMC-7721 and BEL-7402 cells while that total YAP1 expression did not change. (Figure 3A, 3B). We then detected LATS1 and YAP1 mRNA expression by real-time PCR, it showed that there was no change as well. (Figure 3C). These data suggested that TGF-β1 did not affect the expression of YAP1 and LATS1 mRNA at transcriptional level. YAP activity is determined by its phosphorylation status and cellular localization. YAP phosphorylation induces its translocation from the nucleus to the cytoplasm [18]. YAP phosphorylation status can also be regulated by phosphorylated LATS1. We therefore examined phosphorylated LATS1 at Ser909 and YAP1 phosphorylation at Ser127 and Ser397 in SMMC7721 cells treated with TGF-β1, respectively. We found that phosphorylation level of YAP1 at Ser397 and LATS1 at Ser909 increased in a time-dependent manner following incubation with TGF-β1, while phosphorylated YAP1 at Ser127 only increased at 1 and 2 hours (Figure 3D). We also examined the cellular localization of YAP using immunofluorescence staining. Consistent with the increased phosphorylation of YAP1, TGF-β1 treatment induced YAP1 translocation from the nucleus to the cytoplasm after 48 hours (Figure 3E). These findings indicate that one the key mechanisms of TGF-β1 may involve regulation of the Hippo signaling pathway by directly controlling YAP1 phosphorylation and cellular localization, thereby inhibiting the growth of HCC cells.

### TGF-β1 inhibits the growth of HCC cells by regulating the Hippo signaling pathway

**Figure 4:**
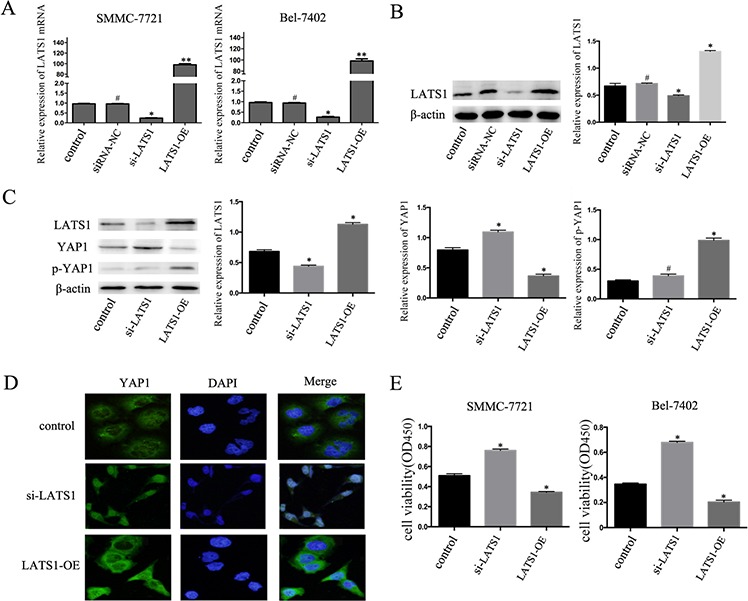
TGF-β1 inhibits the growth of HCC cells by regulating the Hippo signaling pathway **A, B**. The mRNA and protein expression of LATS1 in SMMC-7721 and BEL-7402 cells after transfected by LATS1 siRNA (si-LATS1) or LATS1 overexpressing (LATS1-OE). **C**. Western blotting examined the expression levels of LATS1, YAP1 and p-YAP1 protein in si-LATS1, LATS1-OE, and control transfected in SMMC-7721 cells treated with 5ng/mL TGF-β1 for 48 hours. **D**. immunofluorescent staining of YAP1 **E**. Cell viability was determined by CCK-8 assay. Data are expressed as the mean ± SEM of three independent experiments, *P<0.05.

To further confirm the hypothesis that TGF-β1 inhibits the growth of HCC cells by regulating the Hippo signaling pathway, we transfected HCC cells with LATS1 siRNA and LATS1. At 48 hours after transfection, real-time PCR and western blotting were performed to measure the LATS1 levels in order to evaluate transfection efficiency. (Figure 4A, 4B). And then the cells were treated with TGF-β1 for 48 hours, as shown in Figure 4C, LATS1 special siRNA significantly inhibited YAP1 phosphorylation level and increased YAP1 expression at the protein levels, whereas overexpression of LATS1 inhibited the expression levels of YAP1 protein and increased YAP1 phosphorylation level in SMMC-7721 cells. Besides, LATS1 special siRNA increased YAP1 nuclear translocation compared with those cells overexpression of LATS1 (Figure 4D). Furthermore, inactivation of the Hippo signaling pathway abolished the TGF-β1-induced inhibition of HCC cells (Figure 4E).

In order to illuminate the relationship between Hippo and Smads signaling, we then checked the expression of p-Smad2/3 treated with TGF-β1. The results of western blotting showed that expression of p-Smad2/3 was incresed in a time-dependent manner when treated with TGF-β1 (Supplementary Figure 1A). Besides that, TGF-β1 also induced Smad2/3 translocation from the cytoplasm nucleus to the nucleus (Supplementary Figure 1B). We then use the siRNA to konck down the Smad2/3 expression (Supplementary Figure 1C), cck8 showed that the growth of HCC cells was inhibitied more significantly (Supplementary Figure 1D), it indicated that TGF-β1/smad2/3 promoted the HCC cells growth, while the Hippo pathway inhibited the HCC cells growth and the inhibiton effect was stronger than the promotion effect of TGF-beita/smad2/3. Our data indicate that activation of the Hippo signaling pathway is essential for TGF-β1-induced inhibition of the growth of HCC cells.

### YAP1 shows high expression and nuclear localization in HCC tissues

**Figure 5:**
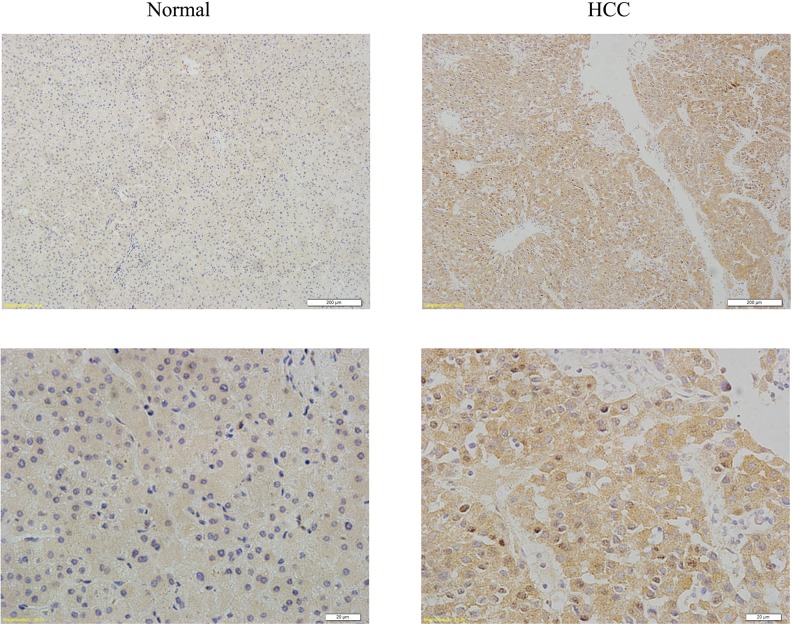
YAP1 shows high expression and nuclear localization in HCC tissues Representative images of normal and HCC tissues immunohistochemical staining with YAP1 antibody. (Original magnification, ×10 and ×40).

We used immunohistochemical staining to evaluate YAP1 expression in HCC tissues. Of the 102 HCC tissues samples, 59.8% (61/102) showed strong YAP1 staining, while normal liver tissue samples showed very weak staining. Moreover, the localization of YAP1 showed stronger nuclear staining (Figure 5). These results indicate that YAP1 is highly expressed and localized in the nucleus in human HCC tissues.

## DISCUSSION

In this study, we reported that TGF-β1 inhibited the growth of HCC cells, upregulated the expression of LATS1 at protein levels, induced nucleocytoplasmic translocation of YAP1, and increased phosphorylation of LATS1 and YAP1 in a time-dependent manner. We also found that overexpression of LATS1 led to a significant decrease in the growth of HCC cells *in vitro*, while downregulation of LATS1 expression promoted HCC cell growth and activated YAP1. Moreover, TGF-β1 inhibited the growth of HCC cells but does not promote apoptosis. Besides, YAP1 is highly expressed and localized in the nucleus in HCC tissues. These findings demonstrate that the Hippo signaling pathway plays an anti-oncogenetic role in the TGF-β1-induced inhibition of the growth of HCC cells.

TGF-β1 has multiple functions in tumors [19–22]. On the one hand, it induces EMT in tumor cells, while on the other hand, it can suppress the invasion and metastasis of cancer cells. Our results showed that TGF-β1 inhibited the growth of HCC cells just by suppressing the HCC cells proliferative capacity. We also examined the expression of Bcl-2 and Bax, which are important targets in the apoptosis process; however, no significant difference was observed in the protein levels of these two proteins. These findings indicate that TGF-β1 could suppress the growth of HCC cells but does not promote cells apoptosis.

Although some studies have shown that YAP1, the effector protein in the Hippo signaling pathway, interacts with TGF-β1 signaling [23–25], other reports that TGF-β1 signaling is independent of the Hippo signaling pathway [26]. In our study, we found that LATS1, the core protein of the Hippo signaling pathway, show increased expression at protein levels, which was induced by TGF-β1. After knock down of LATS1 by transfection of HCC cells with siRNA against LATS1, the TGF-β1-induced inhibition of HCC cell growth was found to be abolished. In contrast, upregulation of LATS1 resulted in a surprising decrease in the growth of HCC cells. Therefore, we have demonstrated that TGF-β1 could induce the activation of the Hippo signaling pathway. Hiemer et al. reported that nuclear YAP1 is necessary for promoting and maintaining the TGF-β1-induced tumorigenic phenotypes in breast cancer cells [27]. Consistent with this, we also found that YAP1 was translocated from the nucleus to the cytoplasm when the HCC cells were treated with TGF-β1, this led to inhibition of HCC cells growth. On another hand, we also found that TGF-β1 increased Smad2/3 phosphorylation expression level and induces nucleocytoplasmic translocation of smad2/3, while after knock out Smad2/3, the growth of HCC cells was significantly inhibited than before. It indicated that TGF-β1/smad2/3 promoted the HCC cells growth, while the Hippo pathway inhibited the HCC cells growth and the inhibiton effect was stronger than the promotion effect of TGF-beita/smad2/3. Taken together, these results demonstrate that TGF-β1 inhibits the growth and development of HCC cells by targeting the Hippo signaling pathway through the regulation of a series of key proteins, especially the nucleocytoplasmic translocation of YAP1.

In conclusion, our findings prove that the Hippo signaling pathway is involved in the TGF-β1-induced inhibition of the growth of HCC cells. TGF-β1 inhibited the growth of HCC cells just by suppressing the HCC cells proliferative capacity, instead of promoting HCC cells apoptosis. TGF-β1 increased LATS1 expression and induced the translocation of YAP1 from the nucleus to the cytoplasm. Overexpression of LATS1 and the nucleocytoplasmic translocation of YAP1 play an anti-oncogenetic role in the occurrence and development of liver cancer. Our findings provide new insight into strategies for liver cancer therapy.

## MATERIALS AND METHODS

### Cell lines and culture

The human HCC cell lines SMMC-7721, Bel-7402, HepG2 and QGY-7703 were purchased from Shanghai Institute of Cell Bank and grown in 1640 containing 10% fetal bovine serum, penicillin (100 units/mL), streptomycin (100 μg/mL), 2 mM glutamine, and 10 mM HEPES buffer at 37°C in a 5% CO_2_ atmosphere.

### RNA extraction and real-time PCR

RNA was extracted using Trizol (Invitrogen, Carlsbad, CA, USA), and real-time PCR was performed using previously described methods [28]. LATS1: forward, 5′-AATTTGGGACGCATCATAAAGCC-3′ and reverse, 5′-TCGTCGAGGATCTTGGTAACTC-3′; YAP1: forward, 5′-TAGCCCTGCGTAGCCAGTTA-3′ and reverse, 5′-TCATGCTTAGTCCACTGTCTGT-3′; 18S was used as control: forward, 5′-GGAGCGAGA TCCCTCCAAAAT-3′ and reverse, 5′-GGCTGTTGTCA TACTTCTCATGG-3′.

### Cell proliferation assay

Used CCK-8 kit (Solarbio, Beijing, China) to evaluated the HCC cells proliferation. HCC cells (1.5 × 10^3^ per well) seeded in 96-well plates were incubated with TGF-β1 for 48 h. About 10 μl of CCK-8 solution was added to each well, and the 96-well plate was incubated at 37°C for 2 h. Then used a multi-label plate reader to measure absorbance at 450 nm (Bio-Rad, Hercules, California USA).

### Colony formation assay

Cells were seeded into 6-well plate at a density of 5 × 10^2^ cells per well and cultured for another fortnight. The colonies were stained with crystal violet and counted under a microscope. All the experiments were performed in triplicate.

### Apoptosis assay

The cell apoptosis was determined by flow cytometer analysis. Cells (2 × 10^5^/well) were seeded in 6-well plate and treated with TGF-β1 for 48 hours and the apoptotic cells were quantified by a FITC-labelled AnnexinV/PI Apoptosis Detection Kit (Invitrogen). All the experiments were performed in triplicate.

### Immunohistochemical staining, western blotting and antibodies

Immunohistochemical staining and western blotting assays were performed using previously described methods [29]. The relative expression of protein was analyzed using Image J software with FracLac plugin java 1.6. Anti-YAP1 rabbit monoclonal antibody and anti-LATS1 rabbit monoclonal antibody were purchased from Proteintech. Anti-phospho-YAP1(Ser127) and anti-phospho-YAP1(Ser397) and anti-phospho-LATS1(S909) and HRP-linked secondary antibodies were purchased from Cell Signaling Technology, China. Anti-β-actin was purchased from TransGen Biotech (Beijing, China). Bcl-2 and Bax rabbit monoclonal antibody were purchased from Boster, China. Smad2/3 and p-Smad2/3 rabbit monclonal antibody were purchased from Proteintech (Wuhan, China).

### Immunofluorescence analysis and reagents

Human recombinant TGF-β1 was purchased from Sigma. Cells were cultured and placed in 6-well plates; they were then washed with PBS and fixed in 4% polyformaldehyde. Following this, the primary antibody YAP1 (1:100) or Smad2/3 (1:100) was added, and the plates were incubated at 4°C overnight. After washing, the fluorescent secondary antibody FITC-labeled secondary antibody (Bioss, BeiJing, China) was added and incubated for 2 h. And then, the cells were counter-stained with DAPI (Bioss, BeiJing, China) and visualized under a confocal microscope.

### RNA interference

All transfections were transfected into cells in six-well plates using Lipofectamine 2000 (Invitrogen), according to the manufacturer’s instructions (GENECHEM, Shanghai, China). Forty-eight hours after transfection, the cells were harvested for real-time PCR or western blotting analysis. The LATS1 siRNA was synthesized by GENECHEM (Shanghai, China) and the sequence was : 5′-GCAAUUGAAUUCAUUAGUAdTdT-3′ and 5′-UA CUAAUGAAUUCAAUUGCdTdT-3′. The Smad2/3 siRNA was synthesized by Sigma (Shanghai, China), the Smad2 siRNA sequence was : 5′-GAGU UCGCCUUCAAUAUGAdTdT-3′ and 5′-UCAUAU UGAAGGCGAACUCdTdT-3′; the Smad3 siRNA sequence was : 5′-CAUGGACCCAGGUUCUCCAd TdT-3′ and 5′-UGGAGAACCUGCGUCCAUGdTdT-3′.

### Statistical analysis

The data analyses were conducted using SPSS17.0 (SPSS Inc., Chicago, IL). Data are presented as the mean ± SD, and statistical analyses were performed using the Student *t*-test or analysis of variance. P-values < 0.05 were considered to indicate statistical significance.

## SUPPLEMENTARY MATERIALS FIGURES


