# Supplementary material for: Transforming growth factor-beta1 suppresses hepatocellular carcinoma proliferation via activation of Hippo signaling
Source: Oncotarget. 2017 Jan 5;8(18):29785–94. doi: 10.18632/oncotarget.14523 (PMC5444703; doi:10.18632/oncotarget.14523)
Supplement: Supplementary file 1 [file oncotarget-08-29785-s001.pdf]

Transforming growth factor-beta1 suppresses hepatocellular carcinoma proliferation via activation of Hippo signaling

SUPPLEMENTARY MATERIALS

SUPPLEMENTARY FIGURE

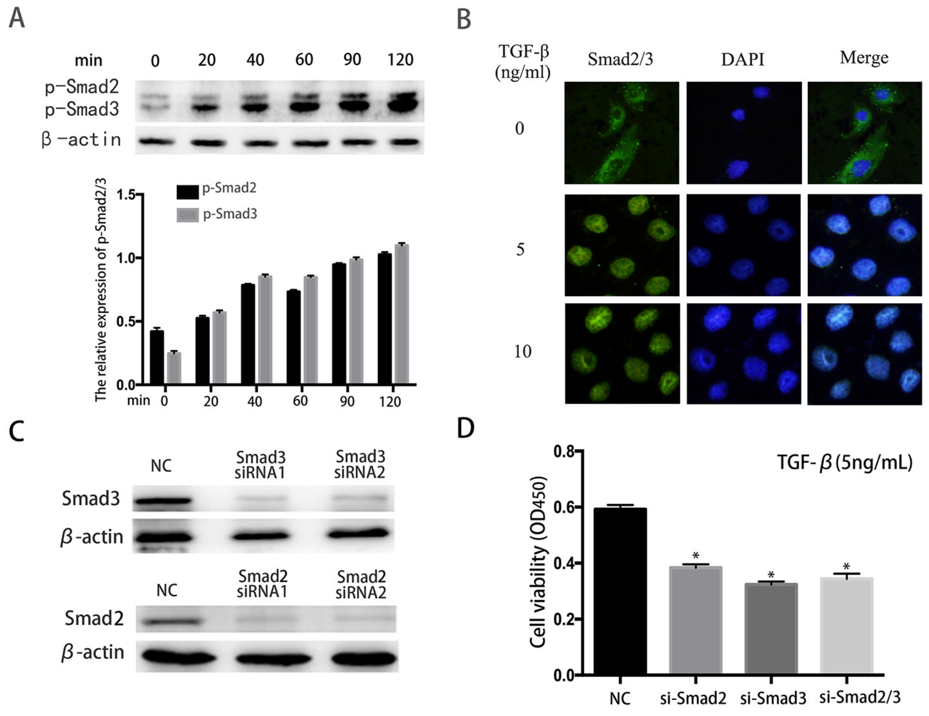

Supplementary Figure 1: A. The protein levels of p-Smad2/3 were measured by western blotting in HCC cells treated with 5ng/mL TGF-β1 for 20min, 40min, 60min, 90min and 120min. **B.** Immunofluorescent staining of Smad2/3 in SMMC-7721 cells treated with 0, 5ng/mL and 10ng/mL TGF-β1 for 48 hours. **C.** and protein expression of Smad2/3 in cells after transfected by Smad2/3 siRNA. **D.** Cell viability was determined by CCK-8 assay. *$P<0.05$.
